# Tetrahedral framework nucleic acids enhance the chondrogenic potential of human umbilical cord mesenchymal stem cells via the PI3K/AKT axis

**DOI:** 10.1093/rb/rbad085

**Published:** 2023-09-15

**Authors:** Liwei Fu, Pinxue Li, Jiang Wu, Yazhe Zheng, Chao Ning, Zhiyao Liao, Xun Yuan, Zhengang Ding, Zhichao Zhang, Xiang Sui, Sirong Shi, Shuyun Liu, Quanyi Guo

**Affiliations:** School of Medicine, Nankai University, Tianjin 300071, People’s Republic of China; Institute of Orthopedics, Chinese PLA General Hospital, Beijing Key Laboratory of Regenerative Medicine in Orthopedics, Key Laboratory of Musculoskeletal Trauma & War Injuries PLA, Beijing 100853, People’s Republic of China; School of Medicine, Nankai University, Tianjin 300071, People’s Republic of China; Institute of Orthopedics, Chinese PLA General Hospital, Beijing Key Laboratory of Regenerative Medicine in Orthopedics, Key Laboratory of Musculoskeletal Trauma & War Injuries PLA, Beijing 100853, People’s Republic of China; Institute of Orthopedics, Chinese PLA General Hospital, Beijing Key Laboratory of Regenerative Medicine in Orthopedics, Key Laboratory of Musculoskeletal Trauma & War Injuries PLA, Beijing 100853, People’s Republic of China; Guizhou Medical University, Guiyang, Guizhou 550004, People’s Republic of China; Institute of Orthopedics, Chinese PLA General Hospital, Beijing Key Laboratory of Regenerative Medicine in Orthopedics, Key Laboratory of Musculoskeletal Trauma & War Injuries PLA, Beijing 100853, People’s Republic of China; Guizhou Medical University, Guiyang, Guizhou 550004, People’s Republic of China; Institute of Orthopedics, Chinese PLA General Hospital, Beijing Key Laboratory of Regenerative Medicine in Orthopedics, Key Laboratory of Musculoskeletal Trauma & War Injuries PLA, Beijing 100853, People’s Republic of China; School of Medicine, Nankai University, Tianjin 300071, People’s Republic of China; Institute of Orthopedics, Chinese PLA General Hospital, Beijing Key Laboratory of Regenerative Medicine in Orthopedics, Key Laboratory of Musculoskeletal Trauma & War Injuries PLA, Beijing 100853, People’s Republic of China; Institute of Orthopedics, Chinese PLA General Hospital, Beijing Key Laboratory of Regenerative Medicine in Orthopedics, Key Laboratory of Musculoskeletal Trauma & War Injuries PLA, Beijing 100853, People’s Republic of China; Guizhou Medical University, Guiyang, Guizhou 550004, People’s Republic of China; Institute of Orthopedics, Chinese PLA General Hospital, Beijing Key Laboratory of Regenerative Medicine in Orthopedics, Key Laboratory of Musculoskeletal Trauma & War Injuries PLA, Beijing 100853, People’s Republic of China; Guizhou Medical University, Guiyang, Guizhou 550004, People’s Republic of China; School of Medicine, Nankai University, Tianjin 300071, People’s Republic of China; Institute of Orthopedics, Chinese PLA General Hospital, Beijing Key Laboratory of Regenerative Medicine in Orthopedics, Key Laboratory of Musculoskeletal Trauma & War Injuries PLA, Beijing 100853, People’s Republic of China; Institute of Orthopedics, Chinese PLA General Hospital, Beijing Key Laboratory of Regenerative Medicine in Orthopedics, Key Laboratory of Musculoskeletal Trauma & War Injuries PLA, Beijing 100853, People’s Republic of China; State Key Laboratory of Oral Diseases, National Clinical Research Center for Oral Diseases, West China Hospital of Stomatology, Sichuan University, Chengdu 610041, People’s Republic of China; Institute of Orthopedics, Chinese PLA General Hospital, Beijing Key Laboratory of Regenerative Medicine in Orthopedics, Key Laboratory of Musculoskeletal Trauma & War Injuries PLA, Beijing 100853, People’s Republic of China; School of Medicine, Nankai University, Tianjin 300071, People’s Republic of China; Institute of Orthopedics, Chinese PLA General Hospital, Beijing Key Laboratory of Regenerative Medicine in Orthopedics, Key Laboratory of Musculoskeletal Trauma & War Injuries PLA, Beijing 100853, People’s Republic of China

**Keywords:** tFNAs, articular cartilage, hUMSCs, chondrogenic differentiation

## Abstract

The field of regenerative medicine faces a notable challenge in terms of the regeneration of articular cartilage. Without proper treatment, it can lead to osteoarthritis. Based on the research findings, human umbilical cord mesenchymal stem cells (hUMSCs) are considered an excellent choice for regenerating cartilage. However, there is still a lack of suitable biomaterials to control their ability to self-renew and differentiate. To address this issue, in this study using tetrahedral framework nucleic acids (tFNAs) as a new method in an *in vitro* culture setting to manage the behaviour of hUMSCs was proposed. Then, the influence of tFNAs on hUMSC proliferation, migration and chondrogenic differentiation was explored by combining bioinformatics methods. In addition, a variety of molecular biology techniques have been used to investigate deep molecular mechanisms. Relevant results demonstrated that tFNAs can affect the transcriptome and multiple signalling pathways of hUMSCs, among which the PI3K/Akt pathway is significantly activated. Furthermore, tFNAs can regulate the expression levels of multiple proteins (GSK3β, RhoA and mTOR) downstream of the PI3K-Akt axis to further enhance cell proliferation, migration and hUMSC chondrogenic differentiation. tFNAs provide new insight into enhancing the chondrogenic potential of hUMSCs, which exhibits promising potential for future utilization within the domains of AC regeneration and clinical treatment.

## Graphical Abstract

**Figure:**
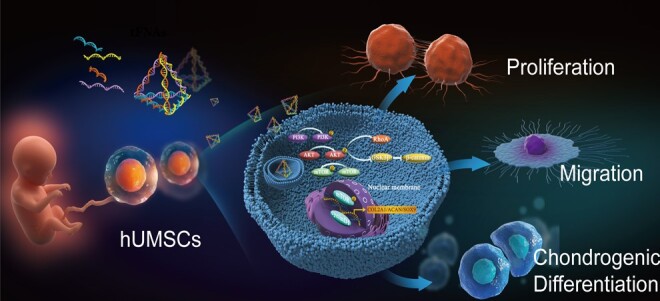

## Introduction

As a kind of vascularless supportive connective tissue, the repair of damaged articular cartilage (AC) has become a main scientific problem faced by the clinic [1, 2]. Currently, the traditional treatment methods for cartilage defects mainly include drug therapy, health education, intra-articular injection and surgical treatment (microfracture, autologous or allogeneic cartilage transplantation and joint replacement), which have made some progress, but many obstacles limit their treatment effectiveness [3, 4]. Advances in AC regeneration and clinical treatment have led to the increasing popularity and promise of using mesenchymal stem cells (MSCs) in treatment strategies for repairing AC defects [5, 6]. Multipotent stem cells known as MSCs exhibit self-renewal and differentiation into different mature cells present in various tissues, including bone marrow (BM), adipose tissue, synovium, blood, umbilical cord and even skin [7]. Specifically, the types of MSCs that are widely used in AC tissue engineering in previous studies are mainly derived from BM, adipose tissue and synovium [8, 9]. In recent years, there has been growing attention given to human umbilical cord mesenchymal stem cells (hUMSCs) because of their significant advantages within the purview of cartilage regeneration [10, 11]. Compared with other common MSCs, hUMSCs have the following several advantages: hUMSCs are derived from the umbilical cord, which does not pose ethical problems; supplies are plentiful and do not harm the donor; the immunogenicity of cells is low and rarely causes immune rejection; and stem cell types are younger and have greater proliferation and differentiation potential [11, 12].

The primary issue when using hUMSCs for the regeneration of AC is the reduced ability to differentiate into chondrocytes, both in laboratory culture settings and in live transplantation environments [13]. With accumulation, hUMSCs will inevitably show a decline in self-renewal and differentiation ability. In particular, when exogenous hUMSCs are transplanted into the joint cavity by injection or tissue engineering, it is often difficult to maintain cell biological functions, including proliferation, migration and chondrogenic differentiation, leading to unsatisfactory AC repair [14]. The cell biological functions of hUMSCs have been stimulated using a variety of bioactive factors and biomaterials to address the aforementioned issues [15–17]. The applications of traditional bioactive factors, including TGF-β3, IGF-1 and BMP-2, are limited because of some shortcomings, such as high extraction cost and single function [15]. Therefore, the search for new ideal bioactive factors to increase the chondrogenic potential of hUMSCs is currently the focus of research within the domains of AC regeneration and clinical treatment.

Tetrahedral framework nucleic acids (tFNAs), a new type of nanoactive material, have attracted much attention because of their great potential within the domains of biomedicine [18, 19]. tFNAs are created through the self-assembly of four predetermined ssDNAs, utilizing base complementary pairing principles. Research has demonstrated that these TFNAs are capable of entering cells through caveolin-mediated endocytosis [20, 21]. tFNAs are widely used in the fields of biological imaging, molecular diagnosis and drug delivery due to the significant advantages of simple synthesis methods, strong edits, great biocompatibility and low immunogenicity [22, 23]. Mounting evidence indicates that tFNAs have a significant impact as regulatory factors in controlling cell behaviour, particularly in the area of tissue regeneration using MSCs [18]. For instance, Shao *et al.* [24] proposed that tFNAs have the ability to modulate the protein expression of the Wnt/β-catenin pathway in adipose-derived mesenchymal stem cells (ADSCs), consequently leading to improved cell proliferation and osteogenic differentiation. In addition, the migration ability of ADSCs can be enhanced by upregulating the expression of RhoA and other proteins [25]. Furthermore, Fu *et al.* [26] demonstrated that tFNAs can affect the biological functions of synovial-derived MSCs (SMSCs) by upregulating multiple signalling pathways and enhancing the *in situ* regeneration of AC *in vivo*. However, the regulatory effects of tFNAs on hUMSC differentiation potential have not been reported.

**Figure 1.**
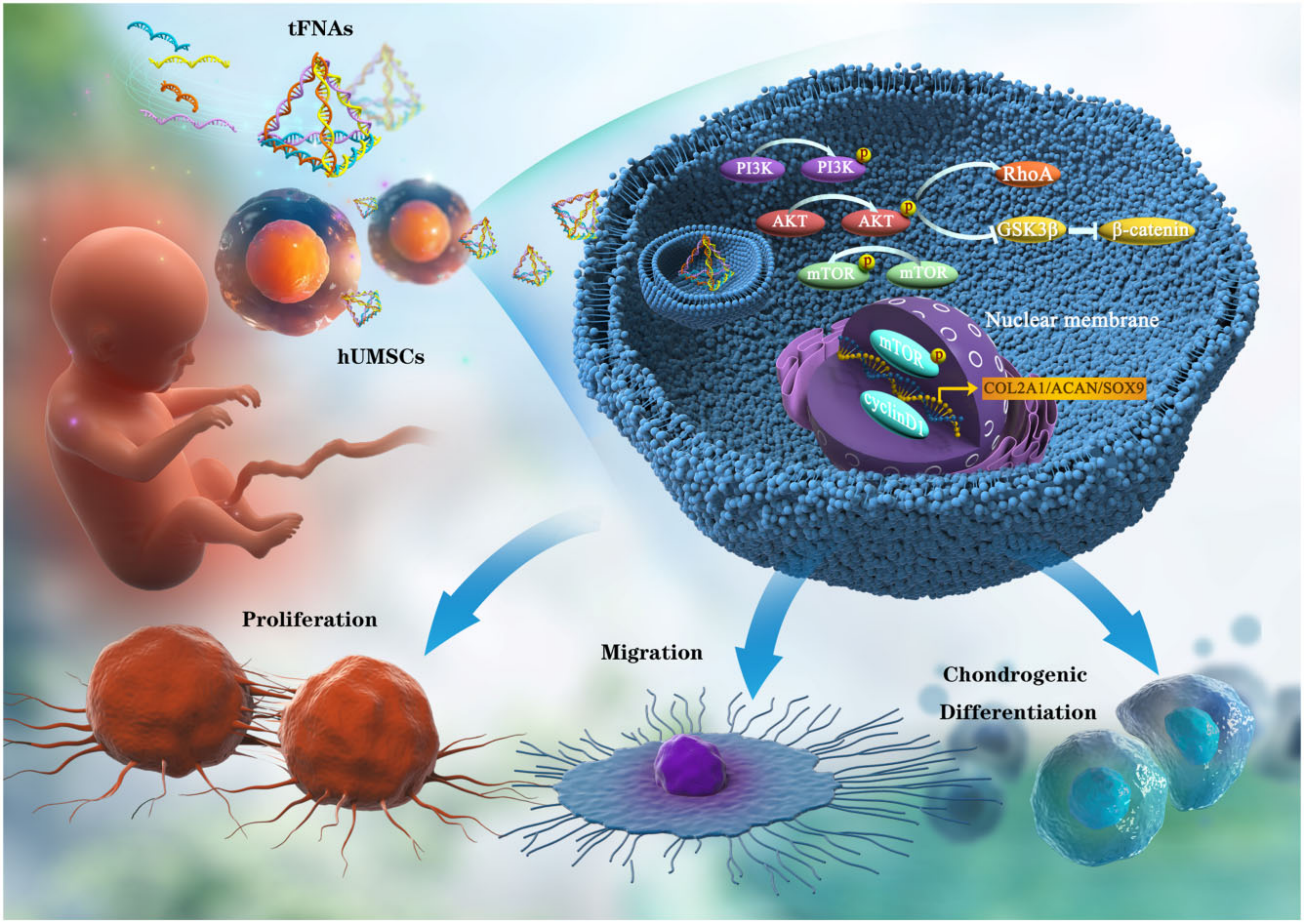
tFNAs improve hUMSC chondrogenic potential through the regulation of the PI3K/AKT pathway.

Hence, in this work, we successfully prepared tFNAs and focused on studying the changes in hUMSC behaviour when exposed to tFNAs. The influence of tFNAs on the hUMSC transcriptome was first analysed by bioinformatics *in vitro*. Then, according to the analysis results, the impact of tFNAs on hUMSC chondrogenic potential (proliferation, migration and chondrogenic differentiation) was explored. Finally, it was confirmed that tFNAs can enhance the chondrogenic potential of hUMSCs by activating the PI3K/Akt axis. Figure 1 provides an overview of the impact of tFNAs. The research shows that tFNAs can be used for the *in vitro* culture of hUMSCs, which is a new application. The results of this study may offer substantial proof for the potential use of tFNAs in UCMSC-based cartilage regeneration and their translation into clinical settings.

## Materials and methods

### Production and representation of tFNAs

tFNA was synthesized by denaturing a mixture of four meticulously designed single-stranded DNAs (ssDNAs) (Supplementary Table S1) at 95°C and subsequently annealing them at 4°C in TM buffer, as per the methodologies outlined in previous research [27]. The molecular weights of the resulting tFNAs were ascertained through polyacrylamide gel electrophoresis (PAGE) and capillary electrophoresis. Moreover, the structural features of tFNA were verified through transmission electron microscopy (TEM), while their morphology was substantiated using atomic force microscopy (AFM). Additionally, dynamic light scattering was utilized to ascertain the average particle diameter and electric charge.

### Cell culture and treatment

The methodology utilized in this study for the isolation and culture of hUMSCs has been previously established [28]. Initially, Wharton’s jelly, a gelatinous substance located within the umbilical cord, was obtained and dissected into fragments 1 mm^3^ in size. These tissue fragments were then affixed onto gauze and placed into a culture flask. To promote optimal cell growth and development, the culture medium was changed every 2–3 days. For more information regarding the experimental procedures, please refer to Supplementary Section S1.1.

### Cellular uptake of tFNAs and mRNA transcriptome sequencing

To assess the internalization of tFNAs by hUMSCs, tFNAs and ssDNA were functionalized with Cyanine5 (Cy5). Following coculturing of the functionalized tFNAs with hUMSCs, fluorescence microscopy (Nikon, Japan) was used to obtain fluorescent images.

The hUMSCs were divided into two groups to conduct subsequent experiments: the (i) blank control group and (ii) tFNA groups incubated with tFNAs (250 nM) based on previous reports [29]. We employed mRNA transcriptome sequencing to detect mRNA changes in hUMSCs under the action of tFNAs. The levels of expression for PI3K/Akt related genes were measured using real-time quantitative polymerase chain reaction (RT–qPCR). Supplementary Section S1.2 contains comprehensive details on the experimental procedures.

### Effects of tFNAs on protein expression in the PI3K/Akt pathway

To demonstrate how tFNAs affect the activity of the PI3K/Akt signalling pathway in hUMSCs, we used immunofluorescence staining to examine the protein expression and intracellular localization of phosphorylated PI3K (p-PI3K), Akt (p-Akt) and mTOR (p-mTOR) after treatment with tFNAs. Additionally, we employed western blotting to analyse the protein expression levels of p-PI3K/PI3K, p-Akt/Akt and p-mTOR/mTOR in hUMSCs with and without tFNA treatment. Supplementary Section S1.3 contains comprehensive details on the experimental procedures.

### Impact of tFNAs on hUMSC proliferation

To evaluate the impact of tFNAs on hUMSC growth, we conducted CCK-8 proliferation assays and EdU proliferation staining assays. To further elucidate the underlying mechanism of tFNA-induced proliferation of hUMSCs, we assessed the expression of glycogen synthase kinase 3β (GSK3β), β-catenin and cyclin D1 via immunofluorescence and western blot analysis. Supplementary Section S1.4 contains comprehensive details on the experimental procedures.

### Effects of tFNAs on hUMSC migration

Transwell chamber experiments and scratch wound healing experiments (using ibidi scratch plugs) were employed to evaluate the influence of tFNA on hUMSC migratory behaviour in both vertical and parallel directions. To further explicate the mechanistic underpinnings of tFNA-mediated migration in hUMSCs, we evaluated the protein expression of RhoA using both immunofluorescence staining and western blotting techniques. Supplementary Section S1.5 contains comprehensive details on the experimental procedures.

### Effects of tFNAs on hUMSC chondrogenic differentiation

To investigate the impact of tFNAs on the chondrogenic differentiation of hUMSCs, hUMSC pellets were constructed *in vitro* based on our previous study [26, 30]. In brief, a total of 4 × 10^5^ hUMSCs were centrifuged at 1300 rpm for 4 min in a 15 ml centrifuge tube, allowing the cells to settle at the bottom of the tube. The centrifuge tube cap was loosely twisted to enable gas exchange, and the cells were subsequently cultured in chondrogenic differentiation basal medium for 24 h. Following the basic culture period, each tube was replaced with chondrogenic differentiation medium containing either 250 nM tFNAs or a blank control. The levels of gene expression for SOX 9, Aggrecan (ACAN) and type II collagen (Col II) were measured using RT–qPCR. Additionally, western blotting was utilized to examine the expression of proteins related to chondrogenesis. The degree of chondrogenesis was assessed at two different time points by haematoxylin-eosin (H&E), Alcian blue, safranin O and Col II immunofluorescence staining. Supplementary Section S1.6 contains comprehensive details on the experimental procedures.

### Statistical analysis

To examine group-wise variations, we conducted one-way ANOVA or Student’s *t*-test using SPSS 18.0 statistical software. Our findings are presented as the mean ± standard deviation, with a significance level of *P < 0.05 considered statistically significant.

## Results

### Successful preparation of tFNAs and cellular uptake

**Figure 2.**
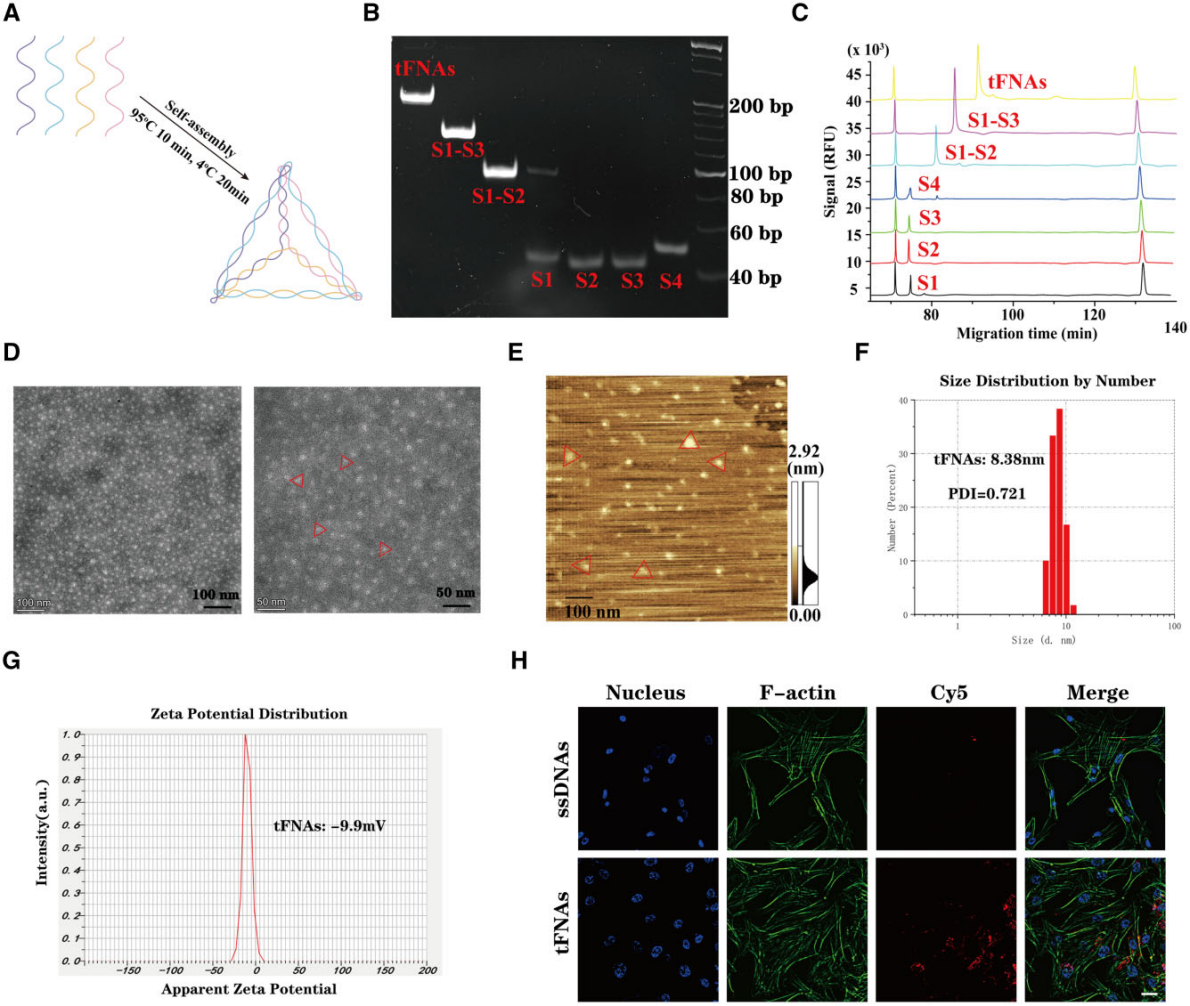
Synthesis, characterization and cellular uptake of tFNAs. (**A**) Schematic diagram of tFNA synthesis. (**B**) PAGE represented the molecular weight of ssDNAs and tFNAs. (**C**) High-performance capillary electrophoresis analysis showed the molecular weights of four ssDNA (S1, S2, S3 and S4) and tFNAs. (**D**) tFNAs were observed by TEM imaging. (**E**) AFM image of tFNAs. (**F**) Size distribution of tFNAs. (**G**) Zeta potential distribution of tFNAs. (**H**) The internalization of tFNAs by hUMSCs. Scale bars are 25 μm.

As shown in Figure 2A, four ssDNAs with specific sequences (Supplementary Table S1) are self-assembled into tFNA through a simple program. The results of PAGE and capillary electrophoresis (Figure 2B and C) showed that four ∼40 bp ssDNA molecules make up a single tFNA (∼160 bp). Furthermore, the micromorphology of tFNAs was scanned using TEM and AFM, revealing that the majority of tFNAs possessed a triangular structure (as depicted in Figure 2D and E). These results confirmed the successful self-assembly of tFNAs. Furthermore, we also examined the size distribution and zeta potential of tFNAs. As shown in Figure 2F, the average particle size of the tFNAs was 8.38 nm, and the polydispersity index representing the size distribution of the nanoparticles was 0.721, indicating that the prepared tFNA nanoparticle system has a good particle size distribution. In addition, the zeta potentials (Figure 2G) suggested that tFNAs are negatively charged on the surface in TM buffer solution, which indicates their stability.

To evaluate the uptake capacity of hUMSCs for ssDNAs and tFNAs, hUMSCs were incubated with 250 nM Cy5-ssDNAs or Cy5-tFNAs. According to Figure 2H, the immunofluorescence images revealed that hUMSCs treated with tFNAs had a considerably stronger Cy5 fluorescence signal than those treated with ssDNAs after a 12-h treatment duration. This suggests that tFNAs can be taken up by hUMSCs with ease and influence relevant biological functions, while ssDNAs were not absorbed in abundance.

### tFNA treatment regulated global gene expression in hUMSCs

**Figure 3.**
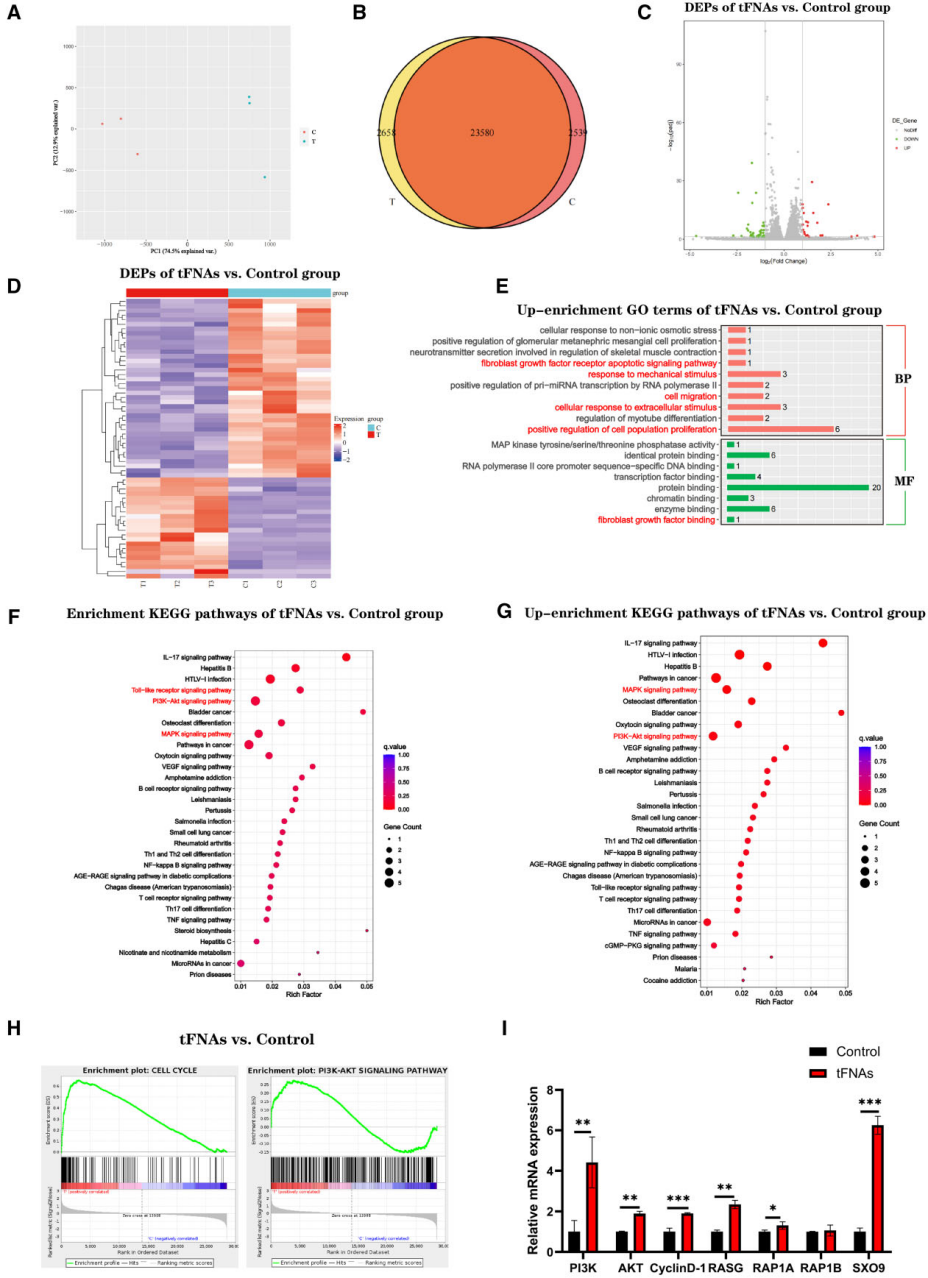
mRNA transcriptome sequencing in hUMSCs under the action of tFNAs. (**A**) PCA of DEGs distributed in the two groups (*n* = 3). (**B**) Venn map of gene expression between different groups. Volcano plot (**C**) and heatmap (**D**) of DEGs between the two groups. (**E**) Up-enrichment GO bar plots of tFNAs versus control group. Enrichment (**F**) and up-enrichment (**G**) KEGG pathways. (**H**) GSEA enrichment analysis of cell cycle and PI3K/Akt signalling pathways between the two groups. (**I**) qRT–PCR analysis of genes associated with PI3K/Akt signalling pathways.

To explore whether culture has priming effects on hUMSCs after exposure to tFNAs, we conducted a transcriptomic comparison using global gene expression analysis to compare the cells in the untreated group with those under tFNA treatment. As shown in Figure 3A and B, the small distance between dots within each group revealed good repetitiveness, while the distances between the control and tFNAs groups were significantly greater. At a fold change cut-off of 2, 61 differentially expressed genes (DEGs) were found in cells treated with tFNAs compared to the control group. Among these genes, 22 genes were up-regulated in tFNA-treated cells, and 39 genes were down-regulated (Figure 3C and D). As show in Figure 3E, Gene Ontology (GO) analysis results showed that compared with the control group, the cell migration in tFNAs treated group, positive regulation of cell population and cellular response to extracellular stimulus related gene enrichment and up-regulation. Further enrichment analysis of KEGG pathway showed that (Figure 3F and G), compared with the control group, DEGs were also up-enriched for functional annotations relating to PI3K/Akt signalling pathway as well as MAPK signalling pathway, which were involved in cellular growth and differentiation. The gene set enrichment analysis (GSEA) results also showed that the cell cycle and PI3K/Akt signalling pathway was enhanced with the addition of tFNAs (Figure 3H). Notably, it was observed that the PI3K-Akt pathway showed significant enrichment, which is known to have vital functions in governing cellular proliferation, differentiation, apoptosis and migration. Therefore, as shown in Figure 3I, we focused on analysis and found that the expression of several important related genes in the PI3K/Akt signalling pathway was up-regulated, and the expression of cyclin D1 and SOX9, an important regulator of cartilage differentiation, was also up-regulated. Finally, we chose the PI3K-Akt signalling pathways to do further studies.

### tFNAs enhanced the phosphorylation level of the PI3K/Akt/mTOR signalling pathway in hUMSCs

**Figure 4.**
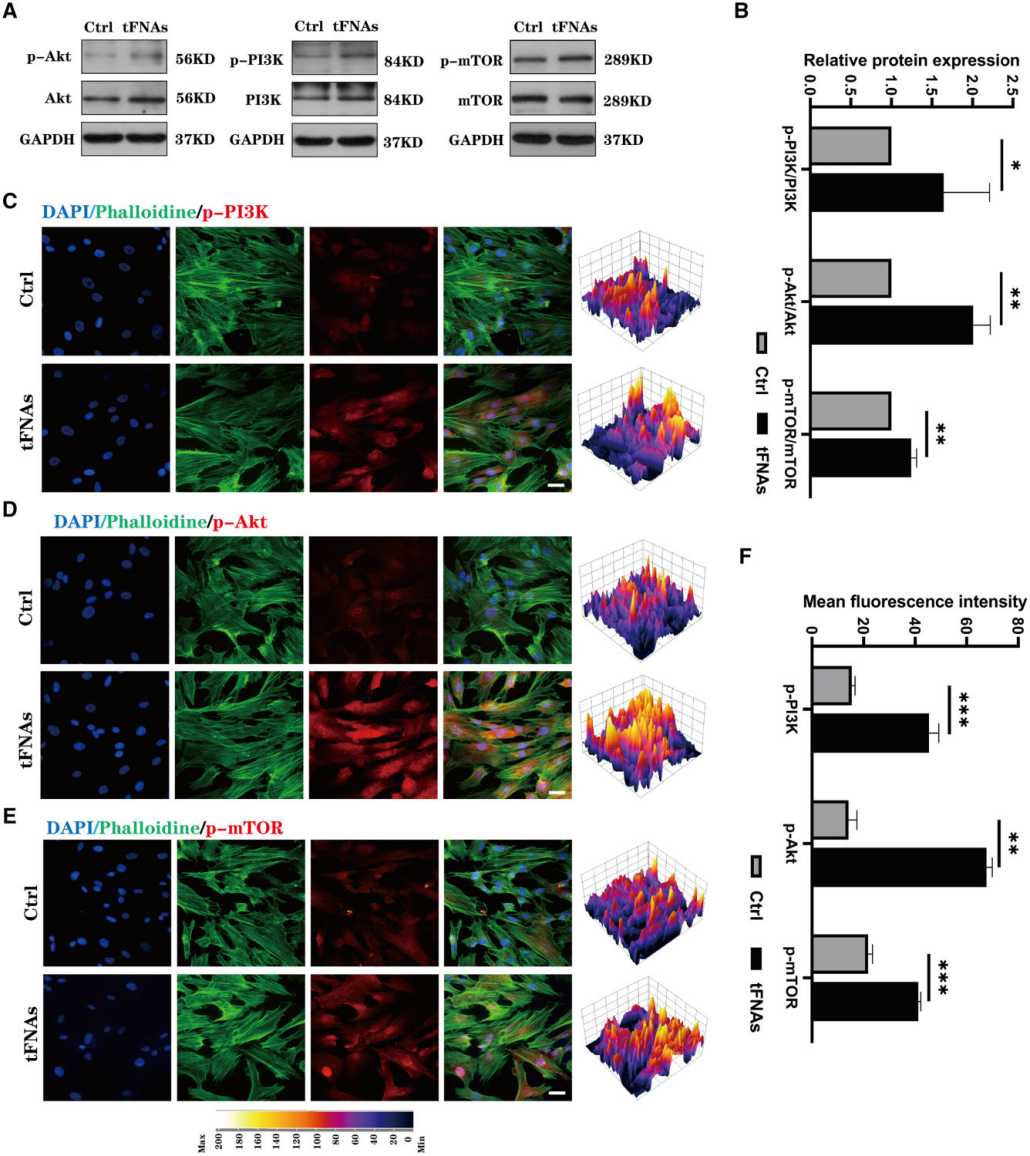
Effect of tFNAs on the activity of the PI3K/Akt signalling pathway of hUMSCs. (**A**) Protein expression of p-PI3K/PI3K, p-Akt/Akt and p-mTOR/mTOR in hUMSCs treated with tFNAs. (**B**) Quantitative analysis of the protein expression levels of p-PI3K/PI3K, p-Akt/Akt and p-mTOR/mTOR. Data are presented as the mean ± SD (*n* = 3). (**C–E**) Immunofluorescence detection of p-PI3K, p-Akt and p-mTOR (cytoskeleton: green, nucleus: blue and protein: red). Scale bars are 25 μm. (**F**) Quantitative analysis of the average optical density of fluorescence images. Statistical analysis: *P < 0.05, **P < 0.01, ***P < 0.001.

To verify the findings of transcriptome sequencing, we employed western blotting and immunofluorescence staining to evaluate the protein expression levels in the PI3K-Akt signalling pathway of hUMSCs after tFNA treatment. Figure 4A and B illustrates that tFNA treatment resulted in an increase in the phosphorylated degrees of PI3K, Akt and mTOR, as highlighted by the western blot results. As mTOR is a necessary protein downstream of the PI3K-Akt signalling pathway, it plays an integral role in MSC proliferation and differentiation. Additionally, immunofluorescence results depicted in Figure 4C–F also confirmed an up-regulation of the phosphorylated levels of three proteins under tFNA treatment. From these findings, it can be concluded that there was a significant increase in the phosphorylation of the PI3K-Akt signalling pathway following hUMSC uptake of large quantities of tFNAs. This can have an impact on cell proliferation, migration, differentiation and other associated functions.

### tFNAs enhanced hUMSC proliferation via the PI3K/AKT/GSK3β axis

**Figure 5.**
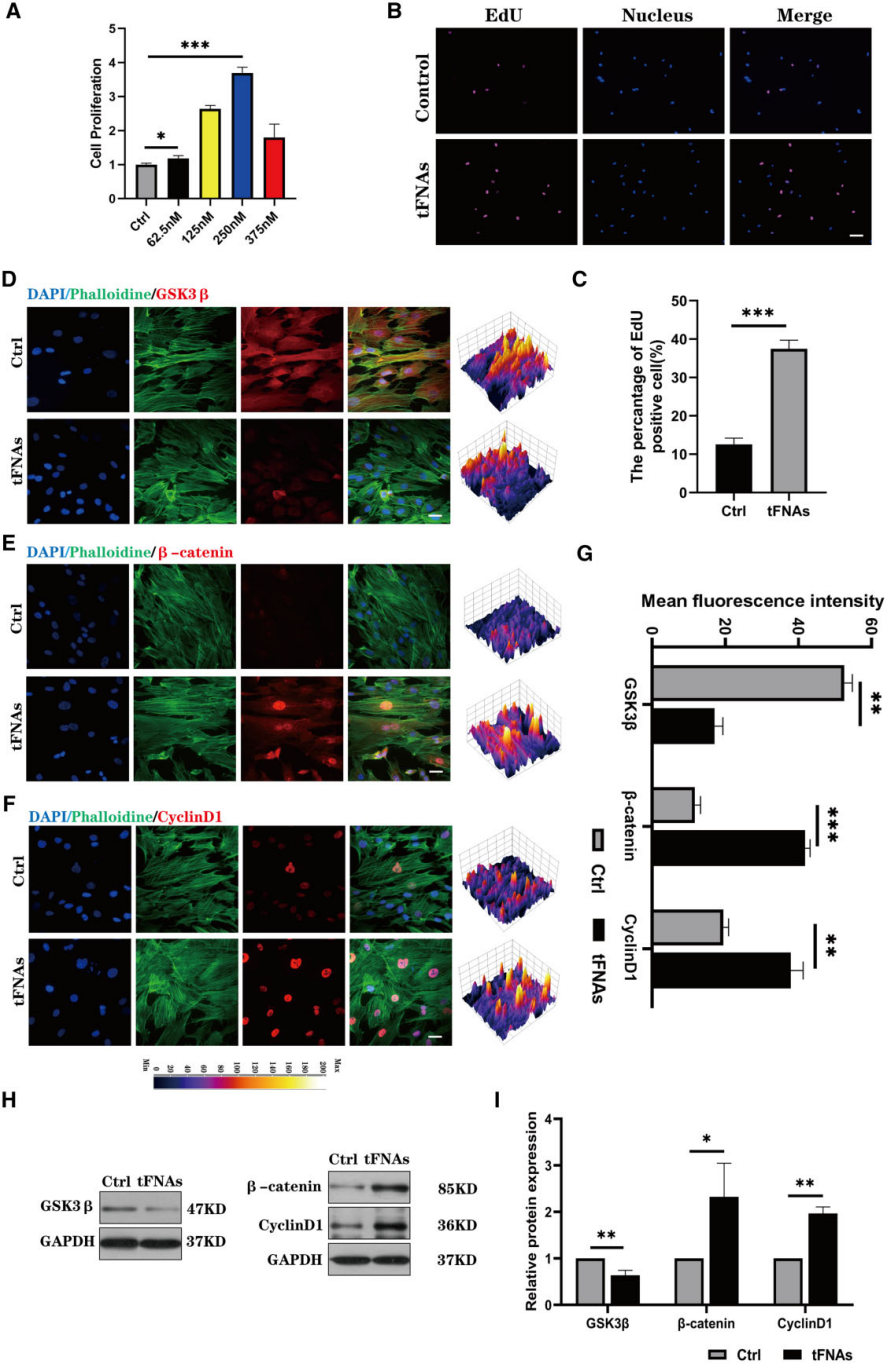
Effect of tFNAs on the proliferation of hUMSCs. (**A**) CCK-8 proliferation assay of hUMSCs treated with tFNAs at different concentrations. (**B**) EdU proliferation staining images of hUMSCs treated with tFNAs (EDU: purple, nucleus: blue). Scale bars are 100 μm. (**C**) Semiquantitative analysis of the EdU proliferation staining assay. Data are presented as the mean ± SD (*n* = 3). (**D–F**) Immunofluorescence detection of GSK3β, β-catenin and cyclin D1 (cytoskeleton: green, nucleus: blue and protein: red). Scale bars are 25 μm. (**G**) Quantitative analysis of the average optical density of fluorescence images. (**H**) Protein expression of GSK3β, β-catenin and cyclin D1 in hUMSCs treated with tFNAs. (**I**) Quantitative analysis of the protein expression levels of GSK3β, β-catenin and cyclin D1. Data are presented as the mean ± SD (*n* = 3). Statistical analysis: *P < 0.05, **P < 0.01, ***P < 0.001.

Studies conducted previously have suggested that the PI3K/Akt pathway contributes significantly to MSC proliferation and is responsible for mediating several factors that contribute to their enhanced proliferation [31]. Therefore, we used two assays to investigate whether tFNAs could increase hUMSC proliferation. Initially, a cell proliferation experiment using CCK8 was conducted to evaluate the effects of tFNA at varying concentrations on hUMSC growth. The findings from the CCK-8 analysis depicted in Figure 5A revealed that tetrahedral treatment significantly enhanced the proliferation potential of hUMSCs, with the optimal concentration being 250 nM. EdU staining was performed to validate the impact of tFNAs on cellular proliferation. The results obtained from EdU staining, including both images and statistical analysis (Figure 5B and C), indicated that in contrast to the control group, hUMSCs cultured in 250 nM tFNAs had more EdU-positive cells, indicating that more cells were in the proliferative cycle after tFNA treatment. In conclusion, tFNAs can significantly enhance the proliferation of hUMSCs, and the optimal working concentration is 250 nM.

Furthermore, we investigated the mechanism by which cell proliferation is enhanced using tFNAs. We found that phosphorylated Akt inhibits GSK3β activity, leading to an accumulation of β-catenin proteins, important transcription factors promoting cell proliferation in the Wnt/β-catenin pathway. To evaluate the expression of upstream and downstream related proteins of β-catenin, including GSK3β, β-catenin and cyclin D1, we utilized immunofluorescence staining and western blotting. The results (Figure 5D–G) showed that β-catenin and cyclin D1 fluorescence intensities were notably increased in hUMSCs treated with tFNA compared to control cells, while the fluorescence intensity of GSK3β was reduced in the tFNA-treated group. Furthermore, western blotting analysis (Figure 5H and I) confirmed the down-regulation of GSK3β expression and up-regulation of β-catenin and cyclin D1 expression levels after tFNA treatment. Thus, these findings suggest that tFNAs promote hUMSC proliferation by activating the PI3K/AKT/GSK3β signalling pathway. Overall, this work provides evidence supporting the proliferative effects of tFNA on hUMSCs.

### tFNAs promoted the migration of hUMSCs through the PI3K/AKT/RhoA axis

**Figure 6.**
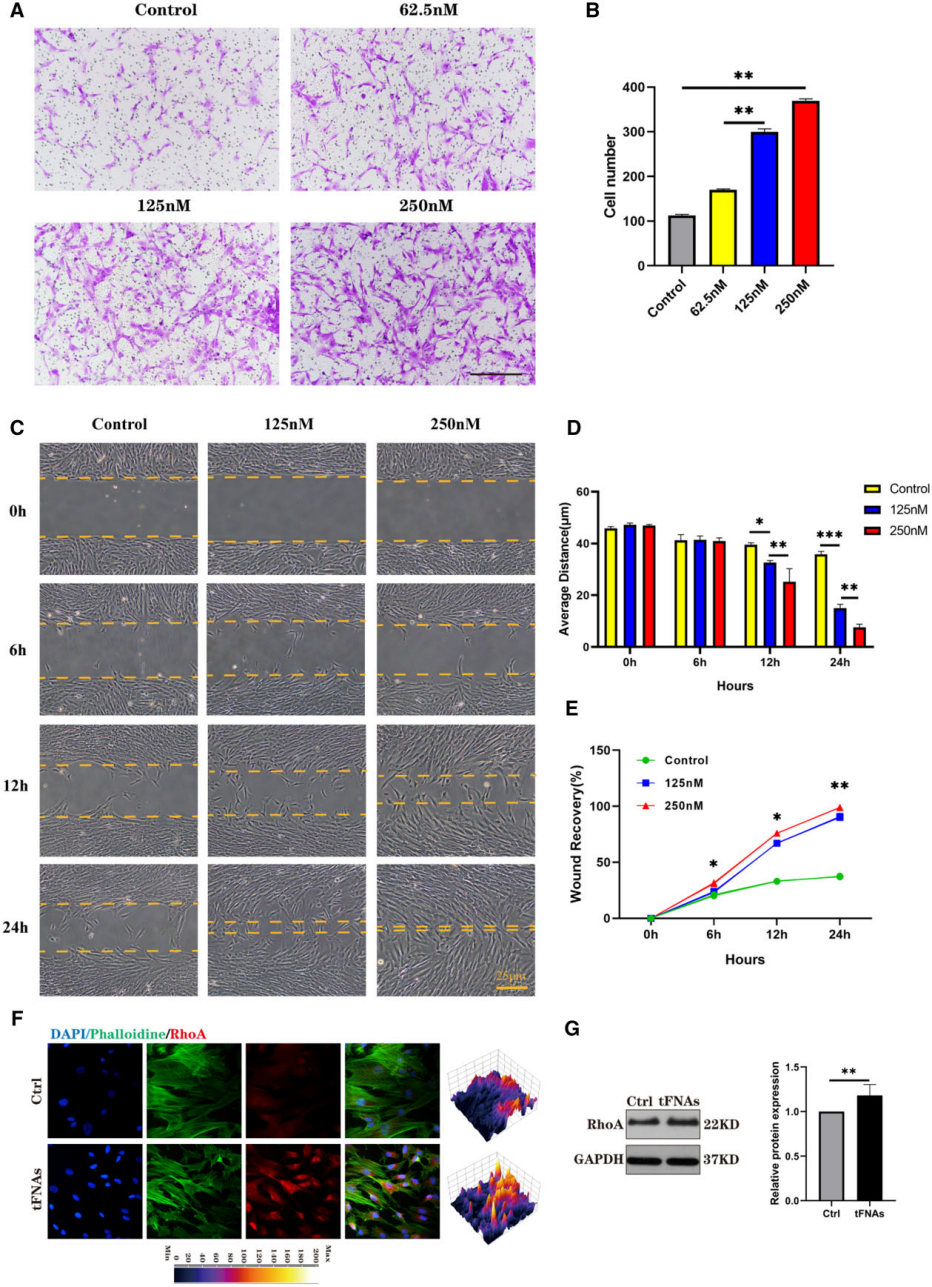
Effect of tFNAs on the migration of hUMSCs. (**A**) Crystal violet staining of hUMSCs treated with tFNAs in transwell chamber experiments. (**B**) Histogram of hUMSCs migrated in the transwell chamber experiment. (**C**) Scratch wound healing experiments demonstrated the effect of different concentrations of tFNAs on hUMSC promotion of migration ability. Scale bars are 250 μm. (**D**) Quantitative analysis of the distance traversed during the scratch wound healing experiment. Data are presented as the mean ± SD (*n* = 3). (**E**) Percentage of wound recovery in the scratch wound healing experiment. Data are presented as the mean ± SD (*n* = 3). (**F**) Immunofluorescence detection of RhoA (cytoskeleton: green, nucleus: blue and protein: red). Scale bars are 25 μm. (**G**) Protein expression of RhoA in hUMSCs and quantitative analysis of the protein expression levels after treatment with tFNAs. Data are presented as the mean ± SD (*n* = 3). Statistical analysis: *P < 0.05, **P < 0.01, ***P < 0.001.

The PI3K/Akt pathway is known to influence cytoskeletal changes and have a crucial impact on cell migration [31]. In MSCs, activation of this pathway has been demonstrated to enhance cell migration [32]. To investigate how this pathway affects the migration of hUMSCs when exposed to tFNAs, transwell and wound healing assays were performed. We evaluated the impact of tFNA on the vertical migration of hUMSCs by treating them with different concentrations of tFNAs and conducting transwell chamber assays to measure their migration. The top surface of the transwell inserts was used to seed hUMSCs followed by incubation with tFNAs for 24 h. Afterwards, crystal violet staining and statistical analysis were conducted on the lower surface of the transwell insert (Figure 6A and B). The results indicated that as the concentration of tFNA increased (0, 62.5, 125 and 250 nM), the number of cells migrating to the lower surface also increased. Moreover, the group exposed to 250 nM tFNA showed the highest number of cells, suggesting that tFNAs promote hUMSC migration in a concentration-dependent manner.

To further examine the effect of tFNAs on the horizontal migration of hUMSCs, a wound healing experiment was carried out as depicted in Figure 6C. As time went by, the group of hUMSCs treated with 250 nM tFNA exhibited faster migration towards the scratch area and subsequently achieved gradual coverage of the scratch. In contrast, there was no significant cell coverage observed in the control group. The vertical distance between the scratched boundaries and the statistical analysis of the covered area were calculated to confirm the observation results (Figure 6D and E). Based on these two experiments, it could be inferred that tFNAs have a notable effect on the promotion of cell migration, with the effect being the best at a concentration of 250 nM.

Furthermore, we also investigated the molecular mechanism of cell migration in detail. According to prior research, it has been suggested that activating the PI3K-Akt axis can increase RhoA expression. This appears to promote cell migration by governing the cytoskeleton as well as retraction forces in the cellular body [31]. To investigate further, we employed immunofluorescence and western blotting techniques to measure the expression level of RhoA. Figure 6F shows that the immunofluorescence staining outcomes highlighted a noticeable boost in the fluorescence intensity of RhoA in hUMSCs treated with tFNA compared to the control group. Moreover, western blotting analysis, as shown in Figure 6G, further confirmed the increased expression level of RhoA after tFNA treatment. In conclusion, combined with the previous experimental results of increased phosphorylation levels of PI3K and Akt, we suggest that tFNAs can enhance the migration of hUMSCs via the PI3K/AKT/RhoA axis.

### tFNAs improved the chondrogenic differentiation of hUMSCs through the PI3K/AKT/mTOR axis

**Figure 7.**
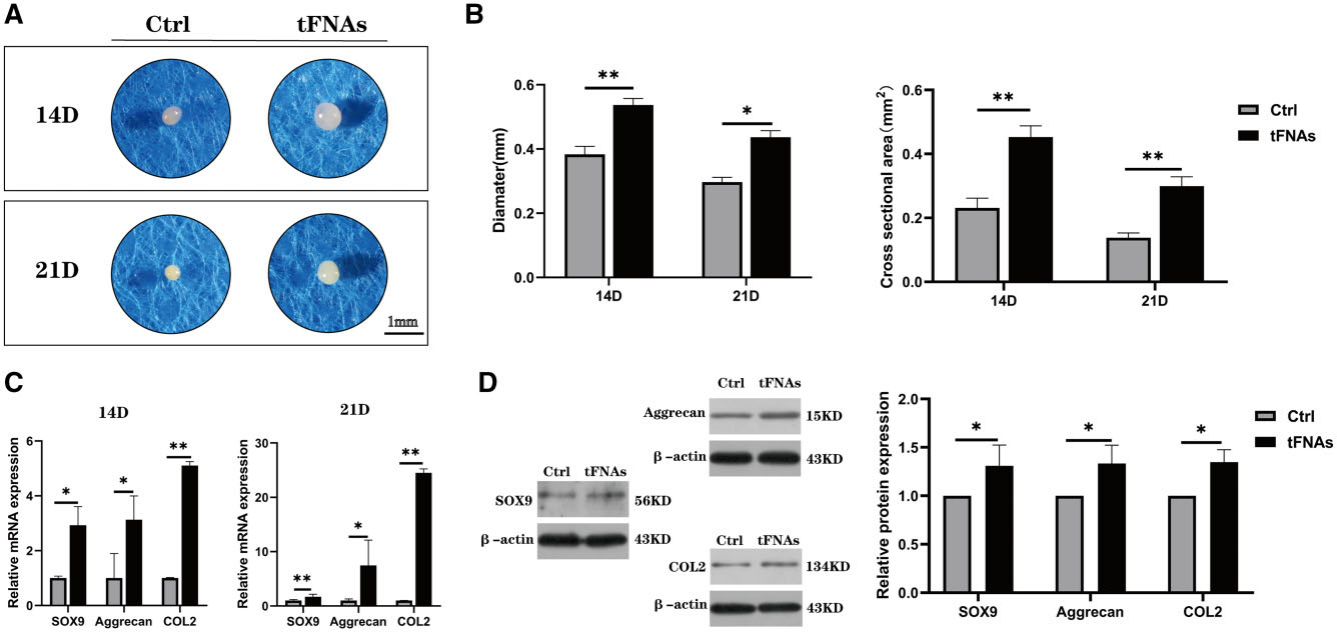
Influence of tFNA on chondrogenic differentiation of hUMSCs. (**A**) Morphological observation of hUMSC pellets. (**B**) Diameter and cross-sectional area of hUMSC pellets at two time points. (**C**) The gene expression levels of SOX-9, ACAN and Col II in hUMSC pellets at 14 and 21 days were quantified via RT–qPCR. Data are presented as the mean ± SD (*n* = 3). (**D**) Protein expression of SOX-9, ACAN and Col II in hUMSC pellets and quantitative analysis of the protein expression levels after treatment with tFNAs. Data are presented as the mean ± SD (*n* = 3). Statistical analysis: *P < 0.05, **P < 0.01, ***P < 0.001.

Research studies have demonstrated that the process of chondrogenesis is significantly influenced by the PI3K/AKT pathway [31]. A pellet culture system based on previous research methods was utilized to investigate the impact of tFNAs on the chondrogenic differentiation of hUMSCs *in vitro*. We added hUMSC pellets into the conventional chondrogenic induction differentiation medium as the control group, and differentiation medium containing 250 nM tFNAs was used as the experimental group. After 14 and 21 days of induced differentiation, the degree of chondrogenic differentiation in the cell pellets was assessed through gross observation, gene and protein quantification and histological staining. As shown in Figure 7A, by gross observation, we found that cell pellets treated with 250 nM tFNAs were larger than those in the control group at both time points. The calculation results of the diameter and cross-sectional area of cell pellets were also consistent with the general view (Figure 7B).

The study utilized qPCR to detect the expression levels of chondrogenesis-specific genes, such as Sox9, ACAN and COL2. Figure 7C indicates that the induced differentiation group treated with 250 nM tFNAs exhibited significant up-regulation of these genes compared to the control group at both time points. To further analyse the protein expression levels of these genes, we extracted proteins from induced cartilage pellets in both groups and conducted western blot analysis. The results presented in Figure 7D supported the qPCR findings, showing an increase in the protein expression levels of Sox9, ACAN and COL2 in pellets after tFNA treatment at 14 and 21 days post-differentiation induction.

**Figure 8.**
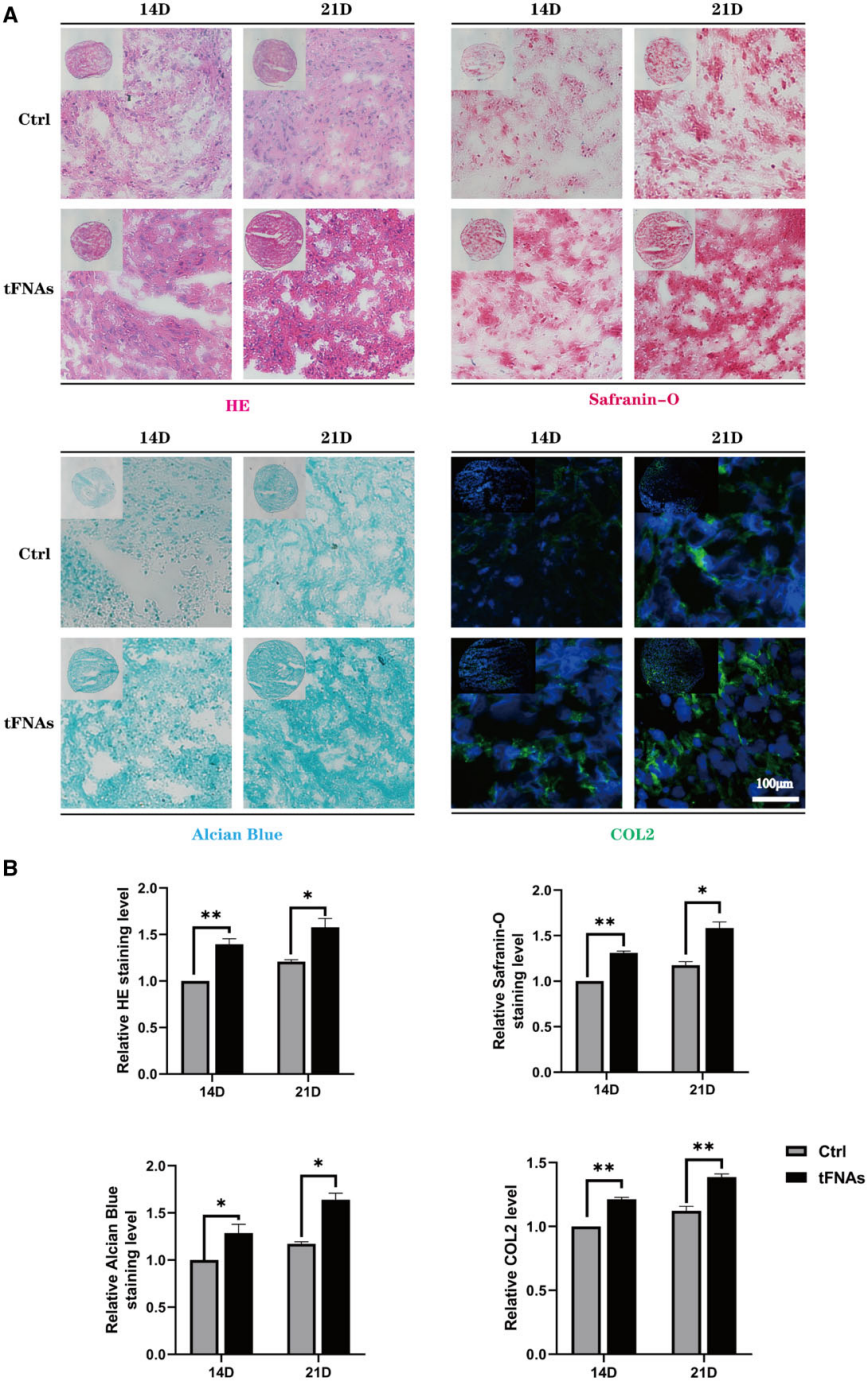
Histomorphological staining of hUMSC pellets. (**A**) The extent of chondrogenesis was evaluated by H&E, Alcian blue, safranin O and Col II immunofluorescence after 14 and 21 days of coculture. (**B**) Semiquantitative analysis of H&E, Alcian blue, safranin O and Col II immunofluorescence. Data are presented as the mean ± SD (*n* = 3). Statistical analysis: *P < 0.05, **P < 0.01, ***P < 0.001.

Additionally, we conducted histomorphological staining to assess the characteristics of the generated cartilage pellets in both groups. The results of H&E staining revealed that after 250 nM treatment, more extracellular stroma was produced in the induced cartilage pellets at both observation time points compared to the control group, as demonstrated in Figure 8A. Then, we used safranin O and Alcian blue staining to detect polysaccharide production in cell pellets. The staining results (Figure 8A and B) showed that in the experimental group, the degree of staining was deeper 14 and 21 days after the induction of differentiation, indicating that tFNA treatment induced the secretion of more cartilage polysaccharide components. Finally, immunofluorescence staining was utilized to assess the Col II levels in cell pellets. The images obtained from the experiment showed a noticeable increase in fluorescence intensity in the experimental group compared to the control group at two distinct time points. These results imply that the utilization of 250 nM tFNAs can augment the creation and buildup of Col II while undergoing chondrogenic differentiation of hUMSCs. In summary, the above findings lend support to the notion that tFNAs can be an effective promoter of chondrogenic differentiation in hUMSCs.

Furthermore, we have demonstrated enhanced phosphorylation levels of mTOR, a key downstream factor in the PI3K/Akt pathway, in ‘tFNAs enhanced the phosphorylation level of the PI3K/Akt/mTOR signalling pathway in hUMSCs’ section. Previous research has revealed that the up-regulation of mTOR signalling could contribute to cartilage development [13]. Based on the findings from the *in vitro* chondrogenic differentiation study mentioned earlier, it can be inferred that tFNAs have the ability to improve the chondrogenic differentiation process of hUMSCs through the involvement of the PI3K/AKT/mTOR axis.

## Discussion

The ability of AC to repair itself after injury is significantly limited because of the absence of blood vessels, nerves and the lymphatic system. Without timely treatment, damaged AC will gradually expand and affect the structure of surrounding normal tissues, eventually leading to osteoarthritis (OA) [33]. OA will cause patients to gradually lose joint function, suffer great physical and mental pain, and often become disabled in the terminal stage, which brings a huge burden to both patients and social medical care [34]. It has been shown in recent years that MSC-based treatments hold great potential for AC regeneration. Among the most prevalent types of MSCs (ADSCs and SMSCs), hUMSCs offer several benefits, including lower immunogenicity, greater proliferation and differentiation capacity and better availability [11, 35]. However, whether cultured *in vitro* or transplanted *in vivo*, stimulation of the chondrogenic potential of hUMSCs has always been a focus of cartilage regeneration research. During the regenerative process of cartilage, the chondrogenic potential of hUMSCs is mainly reflected through several biological functions, including proliferation, migration and chondrogenic differentiation. Traditional bioactive factors tend to stimulate the function of hUMSCs in a single way and are limited by cost and dose. Due to the influence of immunogenicity and biocompatibility, the stimulation effect of traditional biomaterials is also not ideal. Therefore, the role of nanoengineering systems in MSC function has attracted wide attention because of their ability to microregulate the environment in which cells grow. As a new nanomaterial, tFNAs have been shown to regulate the function of a multitude of MSCs, such as ADSCs, SMSCs and dental pulp-derived mesenchymal cells [18, 36]. Nevertheless, the regulatory function of tFNAs on hUMSC function and the mechanisms involved are yet to be understood.

In this study, we first successfully prepared tFNAs and observed their size and zeta potential. Recent studies have confirmed that tFNA can be absorbed by cells mainly via caveolin-mediated endocytosis and then influence intracellular signal transduction [37, 38]. Therefore, we used cy5 fluorescently labelled tFNA-treated cells and found that hUMSCs took up many tFNAs. In contrast to previous research methods [39, 40], we did not further directly explore the functional changes in hUMSCs. Instead, we first used transcriptome sequencing to deeply explore the gene changes after tFNA treatment. It can be seen from the experimental results that the biological information of the cells was significantly changed after tFNAs were placed in the culture environment of hUMSCs. Moreover, GO enrichment results showed that the cellular components, molecular functions and biological processes of hUMSCs were affected. Further pathway analysis results showed that many signalling pathways that play an important regulatory role in cell behaviour were significantly changed, among which the most enriched and most widely played pathway was PI3K/Akt. The PI3K/Akt signalling pathway is a key factor in managing cell proliferation, migration and differentiation. Additionally, it is involved in various cellular pathologies [31]. By activating this pathway, the efficacy of MSCs in tissue engineering and regenerative therapy can be increased. Numerous growth factors, including vascular endothelial growth factor and fibroblast growth factor, are closely related to this pathway. Based on western blotting and immunofluorescence staining results, it can be preliminarily inferred that tFNAs may modify the biological functions of hUMSCs by increasing the levels of phosphorylated key proteins within the PI3K/Akt pathway.

hUMSCs must proliferate to successfully regenerate AC. In addition to the enrichment of hUMSCs through proliferation during *in vitro* culture, poor therapeutic effects after *in vivo* cell transplantation are often caused by a low proliferation rate [11]. Activation of the PI3K/Akt pathway has been shown to be a key factor in MSC proliferation. Moreover, Jang *et al.* [41] found that prostaglandin E2 can reduce the activity of GSK3β by phosphorylating Akt in MSCs from human umbilical cord blood, resulting in the accumulation of β-catenin proteins, which is a transcription factor that can enhance cell proliferation. Therefore, based on the results of the cell proliferation assay, western blotting and immunofluorescence staining, we found that tFNA promoted hUMSC proliferation through the PI3K/AKT/GSK3β axis. During the *in vivo* repair of AC, MSCs must be moved to the defect to function properly. Some PI3K/Akt pathway activators, such as stromal cell-derived factor-1, have the ability to enhance the migration of MSCs [42]. In addition, studies conducted earlier have indicated that activation of the PI3K/Akt pathway can boost the expression of RhoA, a protein that regulates the cytoskeleton and retraction forces in the cell body. This increased expression of RhoA has been observed to enhance cell migration [25, 43]. Therefore, after exploring the cell migration experiment and molecular mechanism, we revealed that tFNAs can enhance the migration of hUMSCs through the PI3K/AKT/RhoA axis.

Chondrogenic differentiation is the last and most critical step for MSCs to regenerate cartilage tissue. It has been extensively shown that the activation of PI3K/Akt plays a crucial role in chondrogenesis [44]. As an example, the research conducted by Chen *et al.* [13] suggested that irisin had the capacity to improve the chondrogenic differentiation of hUMSCs by activating the PI3K/AKT/mTOR pathway. In light of this finding, we decided to conduct an experiment using a cell pellet system to study the effect of tFNAs on the chondrogenic differentiation of hUMSCs. Pellet aggregate culture represents a promising approach for cartilage tissue engineering, as it can be employed as a foundation for scaffold-free tissue engineering, integrated with hydrogels for scaffold-based tissue engineering, and demonstrates potential for *in vivo* cartilage regeneration [30]. Within the scope of this investigation, pellet aggregate culture serves as a means to assess cell viability, proliferation and matrix synthesis in response to tFNA conditions. Based on the observation of the size of cartilage particles after chondrogenic-induced differentiation, detection of gene and protein levels and pathological staining results, it can be concluded that tFNAs can promote chondrogenic differentiation of hUMSCs through the PI3K/AKT/mTOR axis.

According to our findings, as a novel nanomaterial, tFNAs can be taken up by hUMSCs and significantly enhance the chondrogenic potential of cells by stimulating the PI3K/Akt pathway. In addition, to further elucidate the effect of tFNAs on the cell migration and differentiation via PI3K/Akt axis, we also employed a mature PI3K/Akt pathway small molecule antagonist GSK690693 (an ATP-competitive pan-AKT inhibitor) to further explore the mechanism in Supplementary File. The results indicated that the promoting effect of tFNAs on migration and chondrogenic differentiation of hUMSCs was inhibited by GSK690693 (Supplementary Figure S1). The advantages of tFNAs are evident when compared to conventional biomaterials and chemotactic agents. tFNAs have a straightforward preparation process, provide extensive action and have low immunogenicity. However, there are still some limitations in the application of tFNAs. First, although tFNAs are more stable than ssDNA, their degradation rate is still too fast compared to protein factors, which affects their utilization efficiency. Second, the deep mechanism of tFNA action on cells is still unclear, and further experimental exploration is needed. Finally, more research data are needed to support the biological safety of tFNAs. Whether they will produce unpredictable genetic interventions in cells will directly affect the clinical translation of tFNAs. In conclusion, we believe that this new nanomaterial can be used for large-scale culture of hUMSCs *in vitro* or to improve the therapeutic effect of hUMSCs transplanted *in vivo*. In future work, we will continue to deeply explore the effects of tFNAs on the immune regulation and paracrine functions of hUMSCs, laying a foundation for clinical transformation applications in AC regeneration.

## Conclusion

In conclusion, our results demonstrated that tFNAs, novel DNA nanomaterials, significantly enhanced the chondrogenic potential of hUMSCs. Mechanistically, tFNAs activate the PI3K/Akt axis after uptake by hUMSCs. Functionally, tFNAs have the ability to enhance the proliferation, migration and chondrogenic differentiation of hUMSCs, which is achieved by upregulating downstream proteins in the PI3K/Akt signalling pathway. To our knowledge, tFNAs were used for hUMSC *in vitro* culture for the first time in this study and proved to be able to further enhance the application value of hUMSCs in AC tissue engineering. We believe that the results of this study may offer substantial proof for the potential use of tFNAs in hUMSCs-based cartilage regeneration and their translation into clinical settings.

## Supplementary Material

rbad085_Supplementary_Data
