# Supplementary material for: Tetrahedral framework nucleic acids enhance the chondrogenic potential of human umbilical cord mesenchymal stem cells via the PI3K/AKT axis
Source: Regen Biomater. 2023 Sep 15;10:rbad085. doi: 10.1093/rb/rbad085 (PMC10560454; doi:10.1093/rb/rbad085)
Supplement: rbad085_Supplementary_Data [file rbad085_supplementary_data.docx]

Supplementary Materials for

Tetrahedral framework nucleic acids enhance chondrogenic potential of human umbilical cord mesenchymal stem cells via PI3K/AKT axis

Liwei Fu^1,2#^, Pinxue Li^1,2#^, Jiang Wu^2,4#^, Yazhe Zheng^2,4^, Chao Ning^2^, Zhiyao Liao^1,2^, Xun Yuan^2,4^, Zhengang Ding^2,4^, Zhichao Zhang^1,2^, Xiang Sui^2^, Sirong Shi^3^*, Shuyun Liu^2^*, Quanyi Guo^1,2^*

1. School of Medicine, Nankai University, Tianjin 300071, People’s Republic of China.
2. Institute of Orthopedics, Chinese PLA General Hospital; Beijing Key Laboratory of Regenerative Medicine in Orthopedics; Key Laboratory of Musculoskeletal Trauma & War Injuries PLA; No. 28 Fuxing Road, Haidian District, Beijing 100853, People’s Republic of China.
3. State Key Laboratory of Oral Diseases, National Clinical Research Center for Oral Diseases, West China Hospital of Stomatology, Sichuan University, Chengdu 610041, People’s Republic of China.
4. Guizhou Medical University, Guiyang 550004, Guizhou Province, People’s Republic of China.

#These authors contributed equally to this work

*Corresponding author:

(1) Quanyi Guo, Email: doctorguo_301@163.com

1. Shuyun Liu, Email: clear_ann@163.com
2. Sirong Shi, Email: sirongshi@scu.edu.cn
3. **Supplementary Materials and Methods**
   1. **Isolation and culture of hUMSCs**

The study received approval from the Ethical Committee of PLA General Hospital, and informed consent was obtained from the patients. Generally, fresh human umbilical cords were obtained from pregnant women who had undergone full-term cesarean section. Wharton’s jelly was meticulously isolated from the umbilical cord and sectioned into 1 mm^3^ pieces under aseptic conditions. The fragments of Wharton’s jelly were seeded onto sterile gauze placed in a cell culture flask, followed by the addition of culture medium containing 15% fetal bovine serum. The flask was then placed in a constant-temperature incubator for culturing, with the culture medium replenished every 3 days. Sub-culturing was performed upon reaching 90% confluence. To ensure cell viability, only cells from passages 3 to 5 were employed in this study.

**1.2. Cellular uptake of tFNAs and mRNA transcriptome sequencing**

To assess the internalization of tFNAs by hUMSCs, we modified tFNAs and ssDNA with cyanine5 (Cy5) and cocultured them with hUMSCs for 12 h. Subsequently, the hUMSCs were rinsed with PBS and fixed with 4% polyoxymethylene solution for 30 min. After that, hUMSCs were stained with 4′,6-diamidino-2-phenylindole (DAPI, 1:250, Life Technologies) for 5 min. Finally, images of all samples were captured with a fluorescence microscope (Nikon, Japan).

We employed mRNA transcriptome sequencing to detect mRNA changes in hUMSCs under the action of tFNAs. Initially, the RNeasy Mini Kit (Cat#74106, Qiagen) was used to extract total RNA from the samples, following the manufacturer's standard operating procedures. The quality of the total RNA was assessed using a NanoDrop ND-2000 spectrophotometer and Agilent Bioanalyzer 4200 (Agilent Technologies, Santa Clara, CA, US), and only the qualified RNA samples were used for subsequent library construction. The rRNA-depleted RNA was fragmented, double-stranded cDNA was synthesized, the second strand was degraded, the 3’ end was repaired, A-tailing was added, and adapters were ligated, followed by an amplification experiment. The constructed library was quantified using a Qubit® 2.0 Fluorometer, and the size was determined using Agilent2100. The qualified library was prepared for Illumina sequencing, and the sequencing strategy was PE150. The sequencing principle is based on sequencing by synthesis (SBS): clusters with DNA templates were generated on a flow cell, and four fluorescently labeled dNTPs, DNA polymerase, and primers were added to amplify the clusters. As each complementary strand is extended in each sequencing cluster, a corresponding fluorescence is released with the addition of each labeled dNTP. The sequencer captures the fluorescence signal, which is converted to a sequencing peak by computer software, providing the sequence information of the target fragments. Finally, the collected information was analyzed.

The expression of genes related to PI3K/Akt signaling pathways was analyzed by a Cell Total RNA Isolation Kit (Foregene, Chengdu, China). The specific experimental steps were the same as 1.6.

**1.3. Effect of tFNAs on the Activity of PI3K/Akt Signaling Pathway of** **hUMSCs**

1.3.1. Western blotting

hUMSCs were transferred to 6-well plates and cultured of different groups for 1 days. Cell samples were digested with trypsin and centrifuged to detect proliferation-related proteins. Cellular protein extraction reagent (Beyotime, Shanghai, China) was used to extract hUMSC proteins. The protein samples were incubated overnight with the following primary antibodies: anti-β-GAPDH (1:5000, Immunoway, TX, USA), anti-p-PI3K/PI3K (1:2000, Abcam, Cambridge, England), anti-p-Akt/Akt (1:1500, Abcam, Cambridge, England) and anti-p-mTOR/mTOR (1:2000, Abcam, Cambridge, England). The samples were then incubated with the secondary antibody (Abcam, Cambridge, England) for 45 min. Subsequently, the protein bands were visualized using enhanced chemiluminescence.

1.3.2. Immunofluorescence staining

To exemplify the impact of tFNAs on the PI3K/Akt signaling pathway activity of hUMSCs, the protein expression and intracellular localization of phosphorylated PI3K (p-PI3K), Akt (p-Akt), and mTOR (p-mTOR) were assessed via immunofluorescence staining post tFNAs treatment. The hUMSCs were transferred to cell slides prepositioned in a 24-well plate and then cultured of different groups for 3 days. hUMSCs were washed with PBS after delivery, and then the samples were fixed with paraformaldehyde for 30 min. Triton X-100 (0.5%) and immune blocking solution (Beyotime, Shanghai, China) were used to permeabilize and block hUMSCs. After 3 washes with PBS, the samples were incubated with anti- p-PI3K (1:200, Abcam, Cambridge, England), anti-p-Akt (1:200, Abcam, Cambridge, England) and anti-p-mTOR (1:200, Abcam, Cambridge, England) overnight at 4 °C. Fluorescent secondary antibody was added and incubated for 2 h. FITC-phalloidin (Beyotime) was used to stain Cytoskeleton. Finally, the nuclei were stained with DAPI (1:1000, Life Technologies, CA, USA). Images were taken by a fluorescence microscope (Nikon, Japan).

**1.4. Effect of tFNAs on the proliferation of hUMSCs**

1.4.1. CCK-8 proliferation assay

Cell Counting Kit-8 (CCK-8) was used to determine the optimal concentration of tFNAs for proliferation. For CCK-8 assays, hUMSCs were seeded into a 96-well plate at a density of 5000 cells per well. After 24 h of hUMSC culture, conventional growth medium was replaced with DMEM/F12 containing different concentrations of tFNAs (62.5 nM, 125 nM, 250 nM and 375 nM) and 1% (v/v) FBS. For the control group, an equal volume of TM buffer was added to the medium. After 24 h of coculture, the solution in the well plate was replaced with fresh medium containing CCK-8 and reincubated for 2 h. Finally, the optical density (OD) of the CCK-8 solution at 450 nm was measured with a microplate analyzer (Beckman, Fullerton, CA).

1.4.2 EDU proliferation staining assay

EDU proliferation staining assay was used to verify the impact of tFNAs on the proliferation of hUMSCs. A Cell Light EdU In Vitro Kit (Riobio, Guangzhou, China) was used for EDU staining. hUMSCs s were inoculated on cell slides placed on 24-well plates in advance. After overnight treatment on conventional growth medium, hUMSCs were treated with tFNAs or vehicle control for 24 h. Then, the medium was replaced with a 50 mM EDU solution. EDU and DNA staining were then performed separately using Apollo and Hoechst according to the manufacturer's procedures. Fluorescence images of samples were taken with a fluorescence microscope (Keyence, Osaka, Japan).

1.4.3. Immunofluorescence staining

To further elucidate the underlying mechanism of tFNAs-induced proliferation of hUMSCs, we assessed the protein expression of β-catenin pathway-related proteins, such as glycogen synthase kinase 3β (GSK3β), β-catenin, and cyclin D1. The hUMSCs were transferred to cell slides prepositioned in a 24-well plate and then cultured of different groups for 3 days. hUMSCs were washed with PBS after delivery, and then the samples were fixed with paraformaldehyde for 30 min. Triton X-100 (0.5%) and immune blocking solution (Beyotime, Shanghai, China) were used to permeabilize and block hUMSCs. After 3 washes with PBS, the samples were incubated with anti-p- GSK3β (1:200, Abcam, Cambridge, England), anti-β-catenin (1:200, Abcam, Cambridge, England) and anti-cyclin D1 (1:200, Abcam, Cambridge, England) overnight at 4 °C. Fluorescent secondary antibody was added and incubated for 2 h. FITC-phalloidin (Beyotime) was used to stain Cytoskeleton. Finally, the nuclei were stained with DAPI (1:1000, Life Technologies, CA, USA). Images were taken by a fluorescence microscope (Nikon, Japan).

1.4.4. Western blotting

hUMSCs were transferred to 6-well plates and cultured of different groups for 1 days. Cell samples were digested with trypsin and centrifuged to detect proliferation-related proteins. Cellular protein extraction reagent (Beyotime, Shanghai, China) was used to extract hUMSC proteins. The protein samples were incubated overnight with the following primary antibodies: anti-β-GAPDH (1:5000, Immunoway, TX, USA), anti-GSK3β (1:2000, Abcam, Cambridge, England), anti-β-catenin (1:1500, Abcam, Cambridge, England) and anti-cyclin D1 (1:2000, Abcam, Cambridge, England). The samples were then incubated with the secondary antibody (Abcam, Cambridge, England) for 45 min. Subsequently, the protein bands were visualized using enhanced chemiluminescence.

**1.5. Effect of tFNAs on the proliferation of hUMSCs**

1.5.1. Vertical cell migration assays (Transwell chamber experiments)

Transwell chamber assays were performed using Transwell permeable plates (Corning, USA) consisting of poly-carbonate Transwell inserts (8 μm pore diameter) and a 24-well plate. The cells (2 × 10^4^) were seeded in the upper half of the insert membrane and cultured with tFNAs for 24 h. We used DMEM/F12 with 5% FBS as a chemoattractant in the lower compartment of the 24-well plate, and in the upper half of the insert membrane, hUMSCs were cultured in DMEM/F12/ tFNAs with 1% FBS. The cells were fixed with 4% paraformaldehyde for 30 min. After the cells were rinsed with PBS, 0.1% crystal violet staining solution was used to stain them for 10 min. Images of migrating hUMSCs in the lower half of each insert were observed by fluorescence microscopy, and the number of cells that migrated to the lower part of the insert was counted in three microscopic views per well. Subsequently, the difference in the mean cell number per well was analyzed. Each group had 3 replicates.

1.5.2 Parallel cell migration (scratch wound healing experiments)

For scratch wound healing experiments, hUMSCs were seeded in a 6-well plate at approximately 1 × 10^5^ cells per well. When the confluence of the seeded hUMSCs in a 6-well plate reached 80%–90%, two crossing linear scratches were generated by a pipette tip. The cells were then cultured in DMEM/F12 with 125 and 250 nM tFNAs and 1% FBS. Wound healing images were acquired after 0, 12 and 24 h, and the size of the scratches was recorded, measured and analyzed.

1.5.3. Immunofluorescence staining

We evaluated the protein expression of RhoA using both immunofluorescence staining and Western blotting techniques. The hUMSCs were transferred to cell slides prepositioned in a 24-well plate and then cultured of different groups for 3 days. hUMSCs were washed with PBS after delivery, and then the samples were fixed with paraformaldehyde for 30 min. Triton X-100 (0.5%) and immune blocking solution (Beyotime, Shanghai, China) were used to permeabilize and block hUMSCs. After 3 washes with PBS, the samples were incubated with anti-RhoA (1:200, Abcam, Cambridge, England) overnight at 4 °C. Fluorescent secondary antibody was added and incubated for 2 h. FITC-phalloidin (Beyotime) was used to stain Cytoskeleton. Finally, the nuclei were stained with DAPI (1:1000, Life Technologies, CA, USA). Images were taken by a fluorescence microscope (Nikon, Japan).

1.5.4. Western blotting

hUMSCs were transferred to 6-well plates and cultured of different groups for 1 days. Cell samples were digested with trypsin and centrifuged to detect proliferation-related proteins. Cellular protein extraction reagent (Beyotime, Shanghai, China) was used to extract hUMSC proteins. The protein samples were incubated overnight with the following primary antibodies: anti-β-GAPDH (1:5000, Immunoway, TX, USA), anti-RhoA (1:2000, Abcam, Cambridge, England). The samples were then incubated with the secondary antibody (Abcam, Cambridge, England) for 45 min. Subsequently, the protein bands were visualized using enhanced chemiluminescence.

**1.6.** **Effect of tFNAs on chondrogenic differentiation of** **hUMSCs**

1.6.1 RT-qPCR

The expression of genes related to the chondrogenic differentiation of pellets of different groups at 14 and 21 days was analyzed by a Cell Total RNA Isolation Kit (Foregene, Chengdu, China). We extracted total RNA from the pellets after 14 and 21 days of culture and converted total RNA to complementary DNA using 5 × RT Master Mix (Toyobo, Osaka, Japan). RT-PCR was performed on a StepOneTM Real-Time PCR system (Applied Biosystems, USA) using 2 × RealStar Green Fast mixture (Genstar, Beijing, China) according to the standard procedure. The primer sequences for Col II, ACAN, SOX 9 and GAPDH are shown in Table S2. To detect the reliability of the primers, we established fusion curves for each reaction system, and there was no nonspecific amplification in the dissolution curves. Relative mRNA expression was normalized to that of the housekeeping gene GAPDH and calculated by using the 2^-ΔΔ^CT method.

1.6.2 Western blotting

After 21 days of culture, pellet samples were digested with trypsin and centrifuged to detect proliferation-related proteins. Cellular protein extraction reagent (Beyotime, Shanghai, China) was used to extract pellet proteins. The protein samples were incubated overnight with the following primary antibodies: anti-β-actin (1:5000, Immunoway, TX, USA), anti-Col II (1:1500, Abcam, Cambridge, England), anti-SOX-9 (1:1500, Abcam, Cambridge, England) and anti-Aggrecan (1:2000, Novus, NY, USA). The samples were then incubated with the secondary antibody (Abcam, Cambridge, England) for 45 min. Subsequently, the protein bands were visualized using enhanced chemiluminescence.

1.6.3. Immunofluorescence staining

We evaluated the protein expression of COL II of hUMSCs pellets using immunofluorescence staining. The pellet slices were washed with PBS after delivery, and then the samples were fixed with paraformaldehyde for 30 min. Triton X-100 (0.5%) and immune blocking solution (Beyotime, Shanghai, China) were used to permeabilize and block. After 3 washes with PBS, the samples were incubated with anti-COL II (1:200, Abcam, Cambridge, England) overnight at 4 °C. Fluorescent secondary antibody was added and incubated for 2 h. Finally, the nuclei were stained with DAPI (1:1000, Life Technologies, CA, USA). Images were taken by a fluorescence microscope (Nikon, Japan).

**1.7** **Effect of tFNAs on migration and chondrogenic differentiation of hUMSCs with the PI3K/Akt pathway small molecule antagonist.**

1.7.1 cell migration assay

Transwell chamber assays were performed using Transwell permeable plates (Corning, USA) consisting of poly-carbonate Transwell inserts (8 μm pore diameter) and a 24-well plate. The experiment was divided into three groups: Control, tFNAs and tFNAs+GSK690693(Abmole, USA)-treated group. The specific experimental steps were the same as 1.5.

1.7.2 cell chondrogenic differentiation

To investigate the impact of tFNAs and GSK690693 on the chondrogenic differentiation of hUMSCs, we used monolayer cell differentiation experiment. In brief, hUMSCs were seeded into a 6-well plate at a density of 10^5^ cells per well. After 24 h of hUMSC culture, conventional growth medium was replaced with chondrogenic differentiation medium for 7 days. The experiment was divided into three groups: Control, tFNAs and tFNAs+GSK690693-treated group. The levels of gene expression for SOX 9, Aggrecan (ACAN), and type II collagen (Col II) were measured using real-time quantitative polymerase chain reaction (RT‒qPCR). The degree of chondrogenesis was assessed by Alcian blue staining.

1.8 Statistical analysis

To examine group-wise variations, we conducted one-way analysis of variance (ANOVA) or Student’s t test using SPSS 18.0 statistical software. The data are presented as mean ± standard deviation (SD), with *p < 0.05 deemed statistically significant.

1. **Supplementary Results**

2.1 tFNAs enhances migration and chondrogenic differentiation by activating the PI3K/Akt pathway.

As shown in Figure S1 below, we employed a mature PI3K/Akt pathway small molecule antagonist GSK690693 (an ATP-competitive pan-AKT inhibitor) to further explore the mechanism. The results of transwell chamber experiment (Figure S1A) showed that compared with the control group, tFNAs treatment significantly promoted the cell migration ability, while the addition of GSK690693 significantly weakened this effect. In addition, the results of 7-day planar single-layer chondrogenic differentiation experiment showed that the promoting effect of tFNAs on chondrogenic differentiation of hUMSCs was also inhibited by GSK690693 (Figure S1B,C). In summary, these results suggest that tFNAs can promote Cell migration and chondrogenic differentiation, which are closely related to the activation of PI3K/Akt pathway.

1. **Supplementary Figures and Tables**


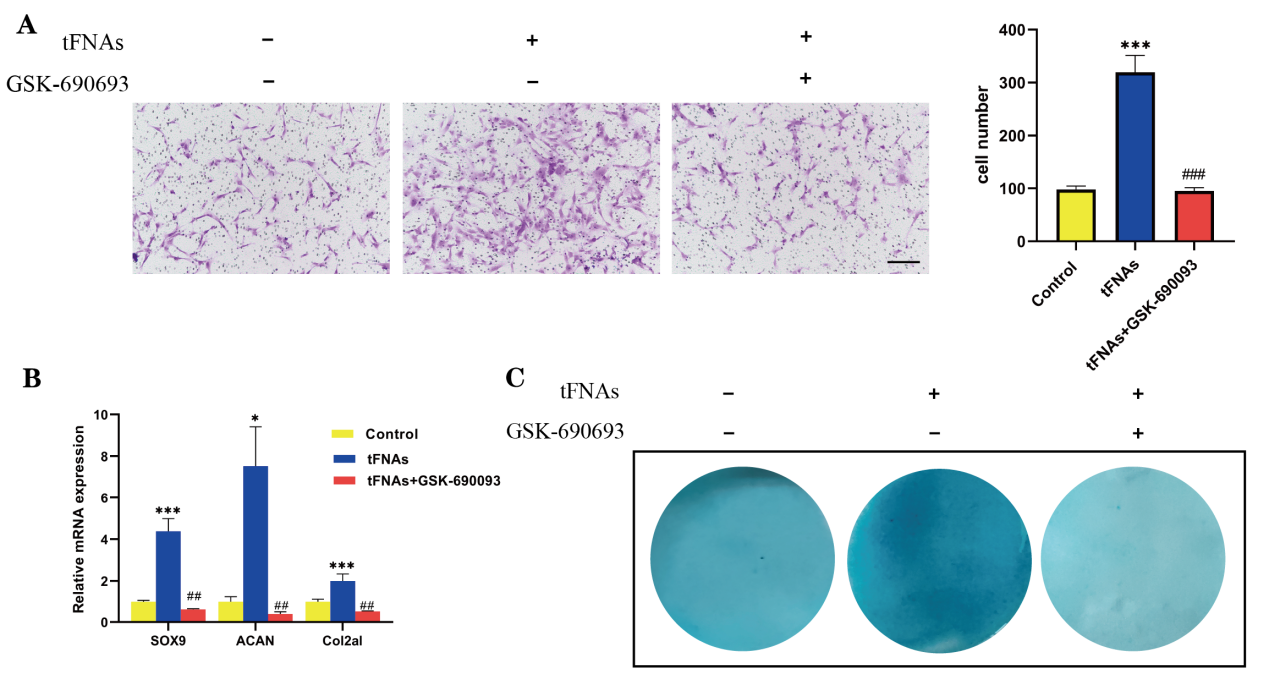


**Figure S1. tFNAs enhances migration and chondrogenic differentiation by activating the PI3K/Akt pathway.** (A) Crystal violet staining and histogram of hUMSCs migrated in the Transwell chamber experiment in control, tFNAs, and tFNAs+GSK690693-treated group, Scale bars are 200 μm. (B) The mRNA expressions of Col2a1, ACAN, and SOX9 were detected by qPCR in control, tFNAs, and tFNAs+GSK690693-treated group. (C) Alcian Blue staining were performed after 7 days of differentiation for monolayer culture in different group. *P<0.05, ***P<0.001 compared with the control group. ##P<0.01, ###P<0.001 compared with the tFNAs-treated group.

**Table S1.** Base sequences of the ssDNAs.

| ssDNA | Base Sequence |
| --- | --- |
| S1 | 5′-ATTTATCACCCGCCATAGTAGACGTATCACCAGGCAGTTGAGACGAACATTCCTAAGTCTGAA-3′ |
| S2 | 5′- ACATGCGAGGGTCCAATACCGACGATTACAGCTTGCTACACGATTCAGACTTAGGAATGTTCG-3′ |
| S3 | 5′-ACTACTATGGCGGGTGATAAAACGTGTAGCAAGCTGTAATCGACGGGAAGAGCATGCCCATCC-3′ |
| S4 | 5′-ACGGTATTGGACCCTCGCATGACTCAACTGCCTGGTGATACGAGGATGGGCATGCTCTTCCCG-3′ |
| Cy5-S1 | 5′Cy5-ATTTATCACCCGCCATAGTAGACGTATCACCAGGCAGTTGAGACGAACATTCCTAAGTCTGAA-3′ |

**Table S2.** Primer sequences for quantitative RT-PCR.

| **Gene** | **Primer** | **Primer Sequence (5' to 3')** |
| --- | --- | --- |
| *GAPDH* | Forward | CCATGTTCGTCATGGGTGTGA |
| *GAPDH* | Reverse | CATGGACTGTGGTCATGAGT |
| *PI3K* | Forward | AACACCGACCTCACAGTTTTT |
| *PI3K* | Reverse | CTCAAGCCACACATTCCACAG |
| *Akt* | Forward | GGAGAGGAAGAGATGGATGCCT |
| *Akt* | Reverse | CCACTTGCCTTCTCTCGAACC |
| *CyclinD1* | Forward | TTCATTTCCAATCCGCCCTCC |
| *CyclinD1* | Reverse | TGACCTGGATAGATGCTGCCA |
| *RAP1A* | Forward | CGTGAGTACAAGCTAGTGGTCC |
| *RAP1A* | Reverse | CCAGGATTTCGAGCATACACTG |
| *RAP1B* | Forward | AGCAAGACAATGGAACAACTGT |
| *RAP1B* | Reverse | TGCCGCACTAGGTCATAAAAG |
| *RASGRP2* | Forward | ACAATCCCGGAAGGACAACTC |
| *RASGRP2* | Reverse | GTCTATGTCGATTAGGCTGCTG |
| *Col II* | Forward | CGTCCAGATGACCTTCCTACG |
| *Col II* | Reverse | TGAGCAGGGCCTTCTTGAG |
| *SOX-9* | Forward | GTACCCGCACTTGCACAAC |
| *SOX-9* | Reverse | TCTCGCTCTCGTTCAGAAGTC |
| *Aggrecan* | Forward | AGAATCCACCACCACCAG |
| *Aggrecan* | Reverse | ATGCTGGTGCTGATGACA |
